# Identification of Prospective Ebola Virus VP35 and VP40 Protein Inhibitors from Myxobacterial Natural Products

**DOI:** 10.3390/biom14060660

**Published:** 2024-06-05

**Authors:** Muhammad Hayat, Tian Gao, Ying Cao, Muhammad Rafiq, Li Zhuo, Yue-Zhong Li

**Affiliations:** 1 State Key Laboratory of Microbial Technology, Institute of Microbial Technology, Shandong University, Qingdao 266237, China; hayatqau89@gmail.com (M.H.); gaotian0420@163.com (T.G.); yingcao0313@163.com (Y.C.); lilab@sdu.edu.cn (Y.-Z.L.); 2 Department of Microbiology, Faculty of Life Sciences and Informatics, Balochistan University of IT, Engineering and Management Sciences, Quetta 87100, Pakistan; 3 Shenzhen Research Institute, Shandong University, Shenzhen 518057, China; 4 Suzhou Research Institute, Shandong University, Suzhou 215123, China

**Keywords:** Ebola virus, VP35 and VP40 proteins, myxobacterial natural products, molecular docking, molecular dynamic simulation

## Abstract

The Ebola virus (EBOV) is a lethal pathogen causing hemorrhagic fever syndrome which remains a global health challenge. In the EBOV, two multifunctional proteins, VP35 and VP40, have significant roles in replication, virion assembly, and budding from the cell and have been identified as druggable targets. In this study, we employed in silico methods comprising molecular docking, molecular dynamic simulations, and pharmacological properties to identify prospective drugs for inhibiting VP35 and VP40 proteins from the myxobacterial bioactive natural product repertoire. Cystobactamid 934-2, Cystobactamid 919-1, and Cittilin A bound firmly to VP35. Meanwhile, 2-Hydroxysorangiadenosine, Enhypyrazinone B, and Sorangiadenosine showed strong binding to the matrix protein VP40. Molecular dynamic simulations revealed that, among these compounds, Cystobactamid 919-1 and 2-Hydroxysorangiadenosine had stable interactions with their respective targets. Similarly, molecular mechanics Poisson–Boltzmann surface area (MMPBSA) calculations indicated close-fitting receptor binding with VP35 or VP40. These two compounds also exhibited good pharmacological properties. In conclusion, we identified Cystobactamid 919-1 and 2-Hydroxysorangiadenosine as potential ligands for EBOV that target VP35 and VP40 proteins. These findings signify an essential step in vitro and in vivo to validate their potential for EBOV inhibition.

## 1. Introduction

Ebola virus (EBOV) is a deadly pathogen that causes hemorrhagic disease [1], known as Ebola virus disease (EVD). It is characterized by gastrointestinal and respiratory issues, hypovolemic shock, and multiple organ failure [2,3]. The average mortality rate is 50–90% [4], posing a potentially significant threat for public health authorities. The epidemic from 2013 to 2016 was the most prevalent, with a mortality rate of 39.52% [5,6]. Recent outbreaks in Guinea and the Democratic Republic of Congo in 2021 and 2022, respectively, as well as the prediction of future episodes across west and central Africa [7], emphasize the need to develop effective drugs to prevent the spread of Ebola virus.

The Ebola virus is a thread-like enveloped RNA virus belonging to the *Filoviridae* family [8]. Its genome is approximately 19 kb and encodes seven structural proteins comprising nucleoprotein (NP), glycoprotein (GP), viral protein (VP24, VP30, VP35), matrix protein (VP40), and RNA-dependent RNA polymerase (L) [8,9,10], as well as non-structural secretory glycoprotein (sGP) and small secretory glycoprotein (ssGP) [11]. The virus primarily targets antigen-presenting cells (APCs), macrophages, and dendritic cells (DCs) for its replication [12]. Among these proteins, GP and L proteins play a role in virus attachment and viral replication, respectively. However, VP35 and VP40 are multifunctional proteins that play a significant role in the EBOV life cycle and pathogenesis, rendering them as plausible targets for drug development. VP35 is a cofactor for the RNA polymerase complex that is critical for RNA synthesis and viral assembly [13]. Furthermore, it suppresses the immune response of the host [14,15] by inhibiting human interferon (IFN-α/β) and RNA interference (RNAi) [16], as well as the dsRNA-dependent protein kinase receptor (PKR), which is vital for IFN synthesis [17]. Similarly, VP40 orchestrates viral assembly and budding [18,19,20,21,22]. Crystallographic analysis revealed that VP40 has two distinct domains, the C-terminal domain (CTD) and the N-terminal domain (NTD) [23], where the CTD plays a role in trafficking to and interacting with the plasma membrane. At the same time, the NTD helps in VP40 dimer formation in the cytoplasm. This strategic localization of the VP40 to the inner leaflet of the plasma membrane is essential for virus-like particle assembly and budding [24,25]. VP35 and VP40 autonomously suppress RNAi, indicating that the EBOV dynamically resists host RNAi during replication [26]. Targeting VP35 offers an optimistic approach to interrupt the EBOV life cycle at multiple stages, while targeting VP40 could hinder viral assembly and stop virus escape from the host cell, hence restricting infection spread.

The Food and Drug Administration (FDA) recently approved two treatments for EVD. The first treatment, Ansuvimab (Ebanga^TM^), blocks viral entry and stops replication [27]. The second treatment, Inmazeb (REGN-EB3), contains three monoclonal antibodies, odesivimab-ebgn, maftivimab and altovimab, which block viral attachment and inhibit cell entry by targeting viral GP [28,29,30]. Although natural products have demonstrated logistic advantages over antibody therapy, no approved anti-Ebola small molecules exist. Another advantage of natural products is their unique properties, such as the vast structural, chemical ecology, and diverse biological activities. For instance, about 50% of FDA-approved drugs are either natural products or their analogues [31]. Recurrent EVD necessitates the screening for probable anti-infective agents using high-throughput screening of natural products from diverse sources.

One promising source of natural products is the myxobacteria—rod-shaped, Gram-negative bacteria that exhibit gliding and swarming motility. These bacteria form desiccation-resistant myxospores under nutrient limitation [32] but germinate in a conducive environment [33]. Myxobacteria commonly reside in soil but have also been isolated from other habitats, including tree bark, seas, freshwater lakes, herbivores’ dung [32], and even extreme environments, such as desert soil [34]. These are well-known producers of natural products, primarily non-ribosomal peptides, polyketides, and their hybrids [35,36,37], which often have novel carbon skeletons with new modes of action [35,38,39]. Myxobacterial compounds have remarkable antiviral activity, with a rare broad-spectrum activity [38,39]. For instance, Noricumazole A from *Sorangium cellulosum* blocks Ebola virus GP-pseudotyped lentiviral vectors [40]. However, detailed investigations of the myxobacterial natural products are required, particularly against viruses. Considering the remarkable success of computer-aided drug discovery, we used a computational approach to identify the potent inhibitors of EBOV VP35 and VP40 from myxobacteria. Furthermore, we aimed to provide a novel insight into the mechanism of the potential hit ligands against both VP35 and VP40 proteins using molecular dynamic simulations and molecular mechanics Poisson–Boltzmann surface area (MMPBSA) calculations. In addition, the drug-likeness of these ligands was predicted via physicochemical, pharmacological, and toxicity profiling.

## 2. Materials and Methods

**Figure 1:**
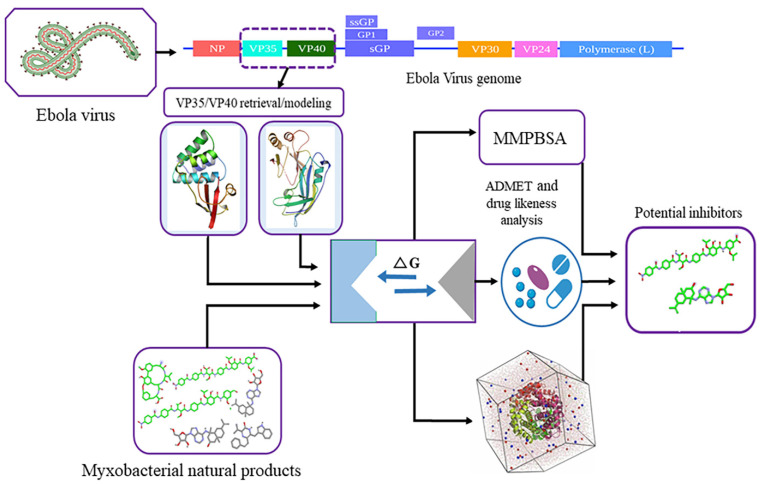
The workflow of this study, including myxobacterial metabolites library preparation, protein retrieval/modeling, molecular docking, molecular dynamic simulation, and ADMET properties.

The current study involves several steps, including preparing the myxobacterial compounds library, retrieving EBOV VP35 and remodeling VP40, conducting ADMET profiling, and performing molecular docking, molecular dynamic simulation, and drug-likeness analysis. These steps are shown in Figure 1.

### 2.1. Retrieval and Structural Evaluation of Proteins

The 3D crystal structure of VP35 (PDB ID, 3FKE, resolution 1.40 Å) was extracted from the RSCB protein databank (https://www.rcsb.org/, accessed 25 December 2023). Upon analysis, the protein structure was completed with no missing residues. However, all 13 experimentally validated structures of the VP40 [41,42] have missing residues; for instance, the VP40 protein structures 1ES6 and 3TCQ, with good resolution of 2.0 Å and 1.6 Å, respectively, both have missing residues as visualized using PyMOL (2.5.2 version).

### 2.2. Remodeling of VP40 Protein

The NTD of VP40 plays a significant role in oligomerization and virus-like particle (VLP) production. We extracted NTD sequences of VP40 from the Mayinga-76, Kikwit-95, and Gabon-94 strains using UniProtKB ID numbers Q05128, Q77DJ6, and Q2PDK5, respectively. Our investigations revealed that these strains have identical NTD sequences. We remodeled the protein using I-TASSER [43,44,45] and Swiss-modeling [46], with 1es6.1.A and 3TCQ as templates. The best I-TASSER model was selected based on the high confidence score, while the best one from SWISS modeling was selected based on qualitative model energy analysis (Qmean). Structural analysis of the top two models revealed that I-TASSER had a more reasonable fold for the missing residues and was selected for further study. Before the molecular docking studies, GROMACS 5.1.5 minimized the energy potential and stabilized the structure. Ramachandran plotting using the VADAR online server (http://vadar.wishartlab.com/, accessed 28 December 2023) [47] was used to assess the reliability and quality of the protein.

### 2.3. Myxobacterial Natural Product Dataset Preparation

We prepared a small library of 173 myxobacterial natural product datasets. The three-dimensional structures were obtained from the NCBI PubChem database (https://pubchem.ncbi.nlm.nih.gov/, accessed 10 November 2023) [48]. The missing structures were prepared by drawing them in Chem-2D and then converting them into three-dimensional structures, and energy was minimized using Chem-3D [49]. The Avogadro tool was used to optimize the ligands [50], and the compounds were then subjected to molecular docking.

### 2.4. Binding Site Evaluation

The binding sites of both VP35 and VP40 proteins were determined using the Computed Atlas of Structure Surface Topography of proteins (CASTp 3.0), available online (http://sts.bioe.uic.edu/castp/index.html?4jii, accessed 5 January 2024) [51]. CASTp predicted more than 15 binding sites, although the sites with relatively small areas and volumes were eliminated. Binding sites were analyzed and predicted using Chimera 1.12.

### 2.5. Virtual Library Screening

Virtual screening was performed to screen myxobacterial compounds using Auto Dock Vina [52]. The 3D structures of the interferon inhibitory domain of VP35 and the N-terminal domain of the matrix protein VP40 were subjected to AutoDock Vina. The graphical user interface tool AutoDock was used to prepare pdbqt files for protein and ligand and grid box generation (ADT). ADT assigned the protein polar hydrogens, and unified atom Kollman charges, solvation parameters, and fragmental volumes. The prepared file was saved in PDBQT format using AutoDock. The grid was designed according to the targetable site predicted by CASTp with the following dimensions and spacing: center_X 2.9837 Å, Y_center 29.0484 Å, and Z_center 12.2486 Å and size center_X 29.5115 Å, center_Y 35.1614 Å, and Z_center 33.1378 Å for VP35; and center_X 39.5418 Å, Y_center 32.6509 Å, and Z_center 33.4288 Å and size center_X 27.63 Å, center_Y 25.5535 Å, and Z_center 29.2151 Å for VP40. A scoring grid was generated using the ligand structure to reduce the computing time. AutoDock/Vina was used for docking with protein–ligand information and grid box parameters specified in the configuration file. An iterated local search global optimizer was used by AutoDock/Vina [52]. Results with a positional root mean square deviation (RMSD) less than 1.0 were grouped and represented by the result with the most favorable binding free energy. The posture with the lowest binding energy was extracted and matched to the receptor structure for further research.

### 2.6. Molecular Dynamic Simulation Analysis

The Desmond module of the Schrödinger software suite 2022-2 version KNIME (v4.5.1) (Schrödinger, LLC, New York, NY, USA) [53] with the OPLS4 (Optimized Potentials for Liquid Simulations) [54] force field was used for molecular dynamic simulations to analyze the conformational changes in protein dynamic motions. The structures of the VP35 and VP40 proteins of the EBOV were solvated in a simulated triclinic periodic boundary box with an extension of 10 Å from each direction using an explicit solvation model (Monte Carlo equilibrated TIP3P), and a transferable intermolecular potential of 3 points was employed for each system [55]. Lennard–Jones interactions (cut-off = 10) and the SHAKE algorithm were employed to regulate the mobility of all covalent bonds, including hydrogen bonds. Additional counter ions (0.15 M of NaCl) were used to neutralize the system during solvation. The protein models were subjected to energy minimization until a gradient threshold of 25 kcal/mol/Å was achieved at a temperature of 300 K and a pressure of 1 bar via the NPT ensemble class. The production simulation was carried out over a timeframe of 200 ns for each system with trajectories extracted at time intervals of 50 ps. The Particle Mesh Ewald (PME) was used to determine the long-range Columbus interactions. In contrast, the RESPA integrator (a motion integration package) [56] was used to regulate all covalent bonds connected to hydrogen atoms, and the inner time step was two fs throughout the simulation. A cut-off value of 9.0 Å was selected for short-range electrostatic interactions, and to analyze long-range van der Waals (VDW) interactions, a uniform density approximation was chosen as the cut-off value. A Nosé–Hoover thermostat [57] with a relaxation time of 12 ps was used at a temperature of 300 K and 1-atmosphere pressure. The Martyna–Tobias–Klein barostat method [58], with a relaxation time of 12 ps, was used to maintain the conditions during the simulation. Subsequently, the stability of each system was evaluated from the trajectories of the molecular dynamic simulation using the root mean square deviation (RMSD), root mean square fluctuation (RMSF), radius of gyration (Rg), hydrogen bond occupancies, and secondary structure elements (SSE) by Schrödinger 2022-2.

### 2.7. Binding Energy Calculations

The binding free energy describes bimolecular interactions between ligands and proteins. Combining the molecular mechanics Poisson-Boltzmann surface area (MMPBSA) with molecular dynamics allows for the computation of the binding free energy of protein and ligand complexes. The van der Waal, electrostatic, and polar solvation energies, and the solvent accessible surface area (SASA) were added to compute the binding free energy. The MMPBSA binding free energies for ligand–protein were calculated using the script “gmx_mmpbsa” provided by GROMACS. The binding energy was calculated using the following equation: ΔG binding = G complex − (G receptor + G ligand). The “ΔG” is the total binding energy of the complex, and the “G receptor” and “G ligand” represent the free receptor and unbound ligand, respectively.

### 2.8. Computational Pharmacokinetics

The toxicity level determined the success of the drug design and development stages. Advanced computational tools and physiochemical properties aid in analyzing drug-like compounds. The six selected compounds’ absorbance, distribution, metabolism, excretion, and toxicity (ADMET) properties were predicted using SWISS-ADME [59].

## 3. Results

### 3.1. Protein Structure and Binding Sites Prediction of EBOV VP35 and VP40

The experimentally validated 3D structure of VP35 (PDB ID:3FKE) was extracted from the protein databank RSCB PDB (https://www.rcsb.org/, accessed 25 December 2023), with a high resolution of 1.40 Å. Structural analysis revealed a complete structure with no missing residues, providing a substantial foundation for studying the interactions with VP35 and its potential inhibitors. In contrast, there are 13 experimentally validated structures of the EBOV matrix protein VP40 [41,42], all of which contain missing residues; for instance, the VP40 protein structure 1ES6 and 3TCQ have good resolution of 2.0 Å and 1.6 Å, respectively, though they have missing residues at 1–43, 192–201, 207–210, 219–231, 293–299, and 306–326 in the NTD. Visualization via PyMOL showed that these missing residues were close to the loop region and RNA binding site, and could not be overlooked, as these residues could impact the ligands with VP40 protein.

**Figure 2:**
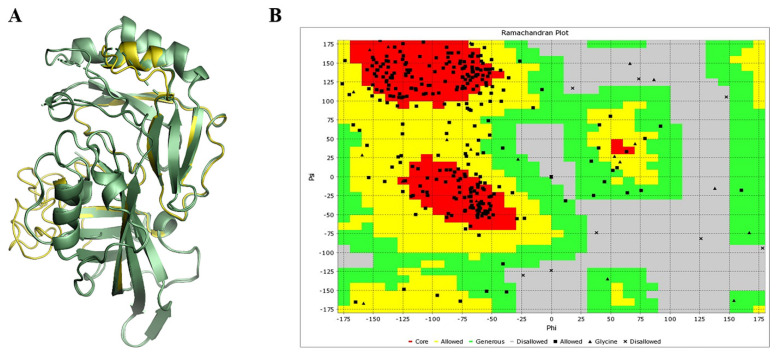
Protein remodeling and Ramachandran plot analysis. (**A**) Remodeled structure of EBOV VP40 protein. The yellow color indicates missing residues of VP40 that were remodeled by I-TASSER, while the olive-green color represents the template 3TCQ protein. (**B**) Ramachandran plot analysis of the remodeled VP40. The red plots show the core residues.

To remodel the structure of the NTD of VP40, its amino acid sequence was retrieved from UniProt using SWISS-MODEL and I-TASSER for reasonable VP40 structure prediction. However, the SWISS-MODEL did not fix all missing residues. I-TASSER generated five protein models with C-scores of −0.70, −1.48, −1.20, −1.86, and −2.99. The C-score typically ranges from −5.0 to 2.0, though a lower value indicates a higher confidence value; hence, the model with a C-score of −0.70 was selected [43,44,45] (Figure 2A). I-TASSER used the experimentally validated crystal structure of the VP40 protein (PDB ID; 3TCQ, 1.6 Å) as a template and had a TM-score of 0.62 ± 0.14. All missing residues were fixed using I-TASSER and more realistically folded compared to the SWISS-MODEL; the selected model has an RMSD value of 0.38, falling within the acceptable range of <2.5 Å [60]. Further a Ramachandran plot (Figure 2B) showed that 95.11% of the residues were present in the favored region, only a few residues were found in the disallowed region, while 0.72% and 0.41% fell in Ramachandran and Rotamer outliers, respectively. Only four C-beta deviations were observed. The above results indicated the excellent quality of the VP40 model.

The interferon inhibitory domain (IID), also known as the CTD of VP35, was retrieved from the PDB database (PDB ID; 3FKE, 1.4 Å). The protein consists of two subdomains: a C-terminal β-sheet and an N-proximal α-helical subdomain, each consisting of approximately 120 residues. A comprehensive analysis of the significant binding pocket prediction of VP35 was performed using the CASTp server and analyzed using Chimera Version 1.12 [61]. A pocket with a good surface area was considered a rational binding pocket for docking analysis. Based on the VP40 model predicted by I-TASSER, CASTp predicted more than 15 binding sites, though the pockets with relatively small areas and volumes were eliminated. As shown in Figure S1 and Table S1, the Pocket 1 of VP35, with a surface of 1155.095 Å2 and 1078.689 Å3, and the Pocket 1 of VP40, with an area of 489.047 Å2 and volume of 298.67 Å3, were considered for further study. The binding site prediction was in agreement with that of the initial Site Finder prediction. The study also proposed higher likeliness of small molecules binding to this site [62].

### 3.2. Molecular Docking Analysis of Myxobacterial Natural Products against EBOV VP35 or VP40

**Figure 3:**
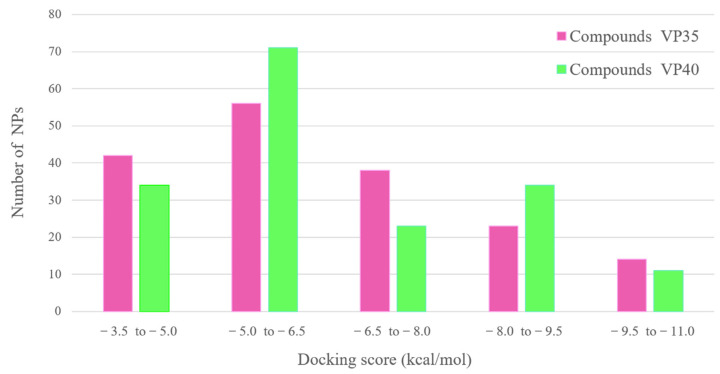
Myxobacterial natural products with their docking score against VP35 and VP40 proteins of EBOV. The y-axis represents the number of natural products (NPs), while the x-axis represents the docking score.

The interaction between ligands and proteins is crucial for understanding biological processes and offers a theoretical basis for developing and identifying new therapeutic targets. This innovative technology enables screening large drug libraries to identify potential drug candidates, which can also significantly reduce the drug discovery time, energy, and cost [63]. We performed the molecular docking of 173 myxobacterial-derived metabolites against EBOV VP35 and VP40 binding pockets. The compounds exhibited a range of binding energies and were grouped into different categories based on their binding energies (Figure 3). The docking results showed that the top three compounds, namely, Cystobactamid-934-2, Cystobactamid 919-1, and Cittilin A, had lower binding energies of −10.5, −10.0, and −9.8 kcal/mol against VP35 protein, while 2-Hydroxysorangiadenosine, Enhypyrazinone B, and Sorangiadenosine had the highest binding affinity with energy scores of −10.9, −10.3, and −8.6 against VP40 (Table S2). These six compounds were selected for further analysis.

**Figure 4:**
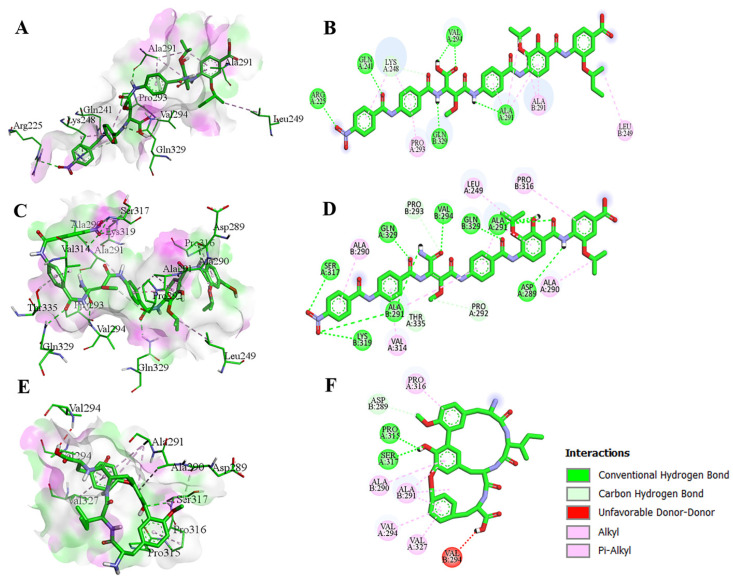
3D and 2D structure of molecular docking analysis of VP35 interacting with Cystobactamid 934-2 (**A**,**B**), Cystobactamid 919-1 (**C**,**D**), and Cittilin A (**E**,**F**).

We evaluated the binding affinity and interaction of Cystobactamid 934-2, Cystobactamid 919-1, and A with VP35. All these compounds interacted with residues from both chains of VP35 through various types of interaction. Cystobactamid 934-2 formed six hydrogen bonds with residues Arg225; A, Gln241; A, Ala291; A, Asp289; B and two bonds with Val294; A, one carbon hydrogen bond with Lys248; A, and four alkyl and pi-alkyl bonds with Ala291; A, Pro293; A, Ala291; B and Leu249; B. Several other residues of the protein chain formed van der Waals interactions (Figure 4A,B). Cystobactamid 919-1 formed 11 hydrogen bonds, creating a single bond with Asp289; A, Ser317; A, Gln329; A, Val294; B, Lys319; B, Gln329; B, two bonds with Ala291; B, and three bonds with Ala291; A. It also formed two carbon hydrogen bonds with Pro292A; A and Pro293; B, and five alkyl and pi-alkyl bonds with Ala290; A, Leu249; A, Ala314; A, Pro316; B, and Ala290; B, while several other residues formed van der Waals interactions (Figure 4C,D). Cittilin A established two hydrogen bonds with Pro315; A and Ser317; A, and one carbon-hydrogen bond with Asp289; B. Hydrophobic interactions were also observed between Cittilin A and Pro316; A, Val294; A, Val237; A, Ala290; B, and Ala291; B (Figure 4E,F).

**Figure 5:**
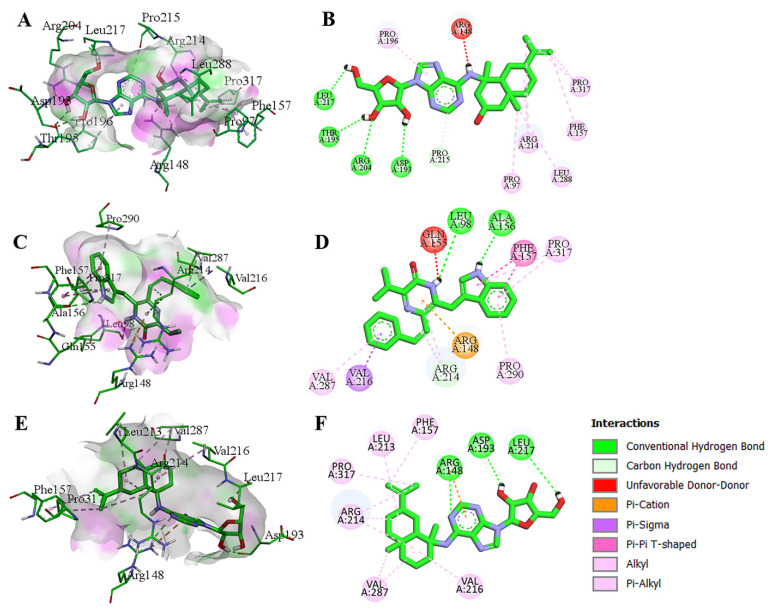
3D and 2D structures of molecular docking analysis of VP40 interacting with 2-Hydroxysorangiadenosine (**A**,**B**), Enhypyrazinone-B (**C**,**D**), and Sorangiadenosine (**E**,**F**).

For the potential inhibitors of VP40, 2-Hydroxysorangiadenosine established four hydrogen bonds (Asp193; A, Thr195; A, Arg204; A, and Leu217; A), one carbon-hydrogen bond (Pro215; A), and six alkyl and pi-alkyl bonds (Pro97; A, Phe157; A, Pro196; A, Arg214; A, Leu288; A, and Pro317; A) (Figure 5A,B). Enhypyrazinone B formed two hydrogen bonds with Leu98; A and Ala156; A, and several hydrophobic interactions, such as Arg148; A, Phe157; A, Arg214; A Val216; A, Val287; A, Pro290; A, and Pro317; A (pi-cation, pi-sigma, pi-pi T-shaped, alkyl, and pi-alkyl interactions) (Figure 5C,D). Similarly, Sorangiadenosine formed three hydrogen bonds with Arg148; A, Asp193; A, and Leu217; A, while Phe157; A, Leu213; A, Arg214; A, Val216; A, Val287; A, and Pro317; A formed hydrophobic interactions (Figure 5E,F). In addition, all the three compounds against VP40 formed several van der Waals interactions.

### 3.3. Molecular Dynamic Simulations of the Main Compounds

Molecular dynamic simulation for 200 nanoseconds (ns) was conducted using the Desmond module of Schrödinger software suite 2022-2 version KNIME (v4.5.1) [53]. The static binding positions of the receptor and ligand complexes at the protein binding site were obtained by molecular docking studies, which can predict the ligand-binding state statically [64]. Molecular dynamic simulation, which calculates atoms’ movement over time using Newton’s classical equation of motion, was used to account for the physiological environment of ligand binding [65,66]. The trajectories were saved every 0.2 ns, and the stability of the simulation was verified by comparing the protein and ligand RMSD over time.

**Figure 6:**
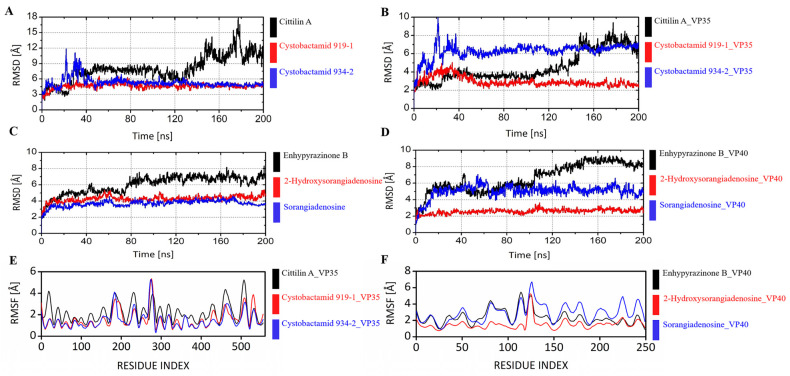
RMSD and RMSF analysis of the protein–compound complex. (**A**,**B**) RMSD analysis of compound alone and compound–VP35 complex. (**C**,**D**) RMSD analysis of compound alone and compound–VP40 complex. RMSF analysis of compound–VP35 complex (**E**) and compound–VP40 complex (**F**). VP35: PDB ID 3FKE. VP40: remodeled structure using I-TASSER.

For the RMSD analysis of ligands and VP35 complexes (Figure 6A,B), the Cystobactamid 934-2_VP35 complex was unstable initially until 45 ns, with a peak of ~20 ns. The complex stabilized after the initial fluctuations, with an average RMSD of 6.27 Å. Cystobactamid 934-2 showed an initial instability in the binding pocket, while stabilizing after 40 ns. The Cittlin A_VP35 complex exhibited a moderate average RMSD of 4.5 Å, with a sudden spike in the RMSD at 40 ns and a gradual increase after 120 ns. The maximum RMSD of 9.40 Å was observed at ~180 ns. The ligand exhibited conformational changes and movement in the binding pocket of VP35, especially at the end of the simulation. However, the Cystobactamid 919-1_VP35 complex had a low average RMSD of 2.95 Å with minor variation at the beginning. The ligand was stable and well accommodated in the binding pocket of VP35 throughout the simulation. The combined RMSD plot of ligands and VP40 complexes is illustrated in Figure 6C,D. The 2-Hydroxysorangiadenosine had a low average RMSD of 4.26 Å, with a slight spike at ~60 ns. It exhibited a stable conformation in the binding pocket of the VP40 protein. A minor peak was observed in the RMSD plot of the complex at ~110 ns, but it attained stability again. Overall, the ligand remained well bound in the receptor during the simulation; however, it slightly changed its initial conformation after 80 ns. The Enhpyrazinone B_VP40 complex showed an elevated average of RMSD 6.0 Å with an increasing deviation at the start of the simulation. A sudden rise in RMSD was observed at ~80 ns, followed by minor fluctuations. Enhpyrazinone B showed a continuous increase in deviation throughout the simulation time. The ligand exhibited movement in the binding pocket, indicating instability. The Sorangiadenosine_VP40 complex had a low average RMSD of 3.68 Å. The complex had a minor deviation at 60–70 ns. However, it stabilized and accommodated well in the binding pocket for the rest of the simulation.

The N- and C-terminal zone loop regions exhibited higher RMSF values, indicating higher flexibility, as confirmed by molecular dynamic trajectories. The lower RMSF values of the binding site residues reflect the stability of the ligand–protein interaction. For the VP35 complexes, Cystobactamid 919-1_VP35 displayed lower RMSF values, whereas Cystobactamid 934-2_VP35 and Cittilin_VP35 showed higher flexibility (Figure 6E). This indicates that Cystobactamid 919-1 binds more intact than the other two compounds. Among the VP40 complexes, 2-Hydroxysorangiadenosine showed minor fluctuations, suggesting a stable interaction (Figure 6F). Sorangiadinosine and Enhydropyrozinone B showed fluctuations and high RMSF values. Therefore, Cystobactamid 919-1 and 2-Hydroxysorangiodinosine were more likely to inhibit the respective proteins.

### 3.4. Protein–Ligand Contacts and MMPBSA Binding Energy Calculations of the Main Compounds

We analyzed the protein–ligand interactions of the receptor–ligand complex using contact histograms. We classified the contacts into hydrogen bond, hydrophobic, ionic, and water-bridge interactions. Hydrogen bonds are crucial for the binding of ligands to proteins. Hydrogen bonding considerably affects drug selectivity, metabolism, and absorption—all vital for drug design [67]. VP35 interacted with ligands through residues from both chains. The residue Gln329 of both chains participated in hydrogen bond formation with all ligands, suggesting its essential role in ligand–protein binding. Gln329 of Chain A formed a hydrogen bond with Cittilin A and Cystobactamid 919-1; however, the Gln329 of Chain B established a hydrogen bond with both Cystobactamid 919-1 and Cystobactamid 934-2. His296; A was involved in a hydrogen bond with Cittilin A, whereas His296; B showed hydrogen bond interaction with Cystobactamid 934-2. We observed that Asp252; B also formed a bond with Cittilin A. Val294; A, Lys335; B, Pro292; B, and Pro316; B of VP35 also contributed to Cystobactamid 919-1 stability during the simulations. For Cystobactamid 934-2, the residues Lys319; A, Ser317; A, Arg225; B, and Arg298; B of VP35 also established hydrogen bonds (Figure S2). In the case of VP40, Ile100 interacted with 2-Hydroxysorangiodenosine and Enhypyrazinone B through a hydrogen bond. Similarly, residues Asp193 and Leu288 formed strong hydrogen bonds with the compounds 2-Hydroxysorangiodenosine and Sorangiodenosine (Figure S3). Other residues of VP40 also participated in hydrogen bonds, such as Leu98 with 2-Hydroxysorangiodenosine, while Asp193 and Pro286 formed a hydrogen bond with Sorangiodenosine. Furthermore, we detected hydrophobic and water-bridge contacts as significant interactive bonds between all ligands and proteins.

Protein and ligand binding free energies are excellent predictors of their interactions. The MMPBSA method accurately depicts the ability of a small molecule to attach to a protein [68]. The binding energies were computed for all complexes utilizing the g_mmpbsa GROMACS tool. The energies of all complexes are listed in Table 1. The total binding energy values for Cystobactamid 919-1, Cystobactamid 934-2, and Cittilin A were −268.16, −214.46, and −191.14 kJ/mol, respectively. Cystobactamid 919-1 exhibited higher energies than the other two compounds for VP35. In the case of the VP40 complexes, 2-Hydroxysorangiadenosine showed the higher total binding energy, i.e., −231.83 kJ/mol, while the total binding energies values were −219.20 and −211.25 kJ/mol for Enhypyrazinone B and Sorangiadenosine, respectively. Therefore, the total binding energy, van der Waals, polar solvation energy, and SASA were favorable for 2-Hydroxysorangiadenosine.

### 3.5. Drug-Likeness and Pharmacokinetics of Bioactive Compounds

Several factors, including toxicity and poor pharmacokinetic properties, lead to failure of drug development. The drug-likeness and pharmacokinetics properties of all compounds were investigated to conclude whether the compounds had favorable absorption, distribution, metabolism, excretion, and toxicity (ADMET) characteristics. Lipinski’s rule of five is significant when developing an orally active medication, which includes molecular weight (MW) of less than 500 g/mol, hydrogen bond acceptors (HBA), rotatable bonds (RB) of less than 10, hydrogen bond donors (HBD), and Log P of less than 5 [69]. Among these compounds, Cystobactamid 934-2, Cystobactamid 919-1, and Cittilin A for the inhibitors of VP35 met Lipinski’s rule of five (Table 2). It is significant in the development of drugs, though there are exceptions. For instance, there are several FDA-approved drugs that violate Lipinski’s rule of five [70] due to their good solubility. ESOL Log S, Ali Log S, and SILICOS-IT Log S were used to predict aqueous solubility [59]. Previous studies reported that Cystobactamid 919-1 significantly inhibited bacterial DNA gyrase, with remarkable potency. The minimum inhibitory concentration was 0.5 µg/mL against Klebsiella pneumoniae to 0.06 µg/mL against Acinetobacter baumannii [71]. Similarly, one of its derivatives played a vital role in reducing bacterial infection in kidneys, lungs, and muscle in an *E. coli* infectious mouse model [38]. All the three potential inhibitors of VP40 conformed to Lipinski’s rule of five (Table 2). Similarly, 2-Hydroxysorangiadenosine did not inhibit any cytochrome P450 (CYP) enzyme. Sorangiadenosine inhibited only the CYP2D6 enzyme, while Enhypyrazinone B inhibited all the CYP enzymes. Although all these inhibitors of VP35 and VP40 presented obvious differences in pharmacological properties, they still have potential druggability.

## 4. Discussion

The EBOV outbreak in 2014–2016 [72] in Africa was the largest in history with a mortality rate of 39.52%. Similarly, the latest outbreaks in the Democratic Republic of Congo and Guinea in 2021 and Uganda in 2022 [73] imply an urgent need for better anti-EBOV drugs. Working with the EBOV is challenging because it is a biosafety level 4 (S4) agent that is life-threatening that requires special laboratory facilities and precautions, which are expensive. Therefore, we used a computational approach to screen a library of 173 myxobacterial compounds for their ability to inhibit two essential EBOV proteins, VP35 and VP40. These proteins are necessary for the virus to evade the host immune system and to replicate effectively. We retrieved the VP35 protein (PDB ID: 3FKE) while remodeling VP40 using I-TASSER, and the protein’s binding site of the protein was predicted using the CASTp online server. In our study, we performed residue-specific docking of myxobacterial compounds to the EBOV VP35 and VP40 structures. We evaluated the binding affinities and interactions of the ligands with binding site residues. Based on binding affinity and hydrogen bonds, all the lead compounds selected against the respective proteins had a remarkable exchange with and high binding affinity to VP35 or VP40 compared to previous studies [73,74,75,76,77]. The hit compounds in our study also had slightly better affinities than Chloroquine and Amodiaquine [78].

VP35 connects the NP and L [79], serving as genome packaging and enabling nucleocapsid formation [8]. It has also been shown to have NTPase and helicase-like functions that are possibly involved in RNA remodeling [80]. Similarly, initial studies revealed that residues in the CTD of EBOV VP35 bind to dsRNA, which is essential for viral polymerase cofactor function [81,82,83,84]. The CTD is also known as an interferon inhibitory domain (IID) [85,86,87]. The binding of the small natural product to the EBOV IID was previously revealed using nuclear magnetic resonance (NMR) and high-resolution X-ray crystallography. The EBOV IID consists of two subdomains: the first basic patch (FBP) and the central basic patch (CBP) [74,87]. FBP acts as a polymerase cofactor and interacts with viral nucleoprotein, whereas CBP binds to dsRNA and inhibits interferon production (INF). The FBP groove is surrounded and lined by various amino acid residues, such as Ala221, Arg225, Gln241, Leu242, Lys248, Lys251, Pro293, Ile295, Ile297, Asp302, Phe302, and Phe328 [88]. Another study also showed that Ala221, Arg225, Ala238, Gln241, Leu242, Val245, Ile246, Lys248, Leu249, Lys251, Ile278, Ile280, Phe287, Pro292, Ile295, His296, Ile297, Asp302, Ala306, Cys307, Pro315, Pro318, Ile320, Asp321, Gly323, Trp324, Val325, Phe328, Leu338, and Ile340 surround the VP35 binding site. These residues may play a crucial role in the conformational dynamics and flexibility of VP35 and are significant for viral and host interaction [77]. Lys319, Arg322, and Lys339 residues in the central basic patch of VP35 [86,89] were identified as significant for dsRNA binding. Similarly, mutations in Arg305, Lys309, and Arg312, substantial as these residues are, could impair the VP35 binding to ds-RNA and DNA and affect IFN-antagonist activity [86]. The binding pocket formed by approximately 20 residues of IID is located between the α-helix and β-sheet subdomains and recognizes dsRNA [74]. Recently in vivo analysis showed that cynarin inhibits VP35-dsRNA with an IC50 value of 8.5 μM, and the docking results revealed that cynarin binds the end-capping site of VP35. The ligand–protein interaction was revealed to be stabilized by numerous hydrogen bonds with the backbone, such as Ala238, Ile340, and Lys339, and with the side-chains of Arg312 and Lys309 [90]. Similarly, another small molecule, known as flavonol myricetin, inhibits VP35 with an IC50 value of 2.7 μM [91]. Our analysis of binding site prediction (Table S1), as well as the molecular docking analysis (Table S2), are consistent with these studies [77,88,89]. The small molecules in our analysis targeting the VP35 IID domain of EBOV could possibly interrupt the viral ability to replicate or show resistance to the immune responses of the host. The small molecules binding either to the key residues of IID like Arg225 or to other residues within this domain might alter the ability of VP35 to contact with dsRNA. This inhibition will not only suppress the production of interferon but also disrupt the polymerase cofactor function. Another major aspect of these compounds could further reduce viral propagation within the host cell. Scientists should focus on the design and synthesis of small molecules to effectively target the IID domain and evaluate their antiviral ability both in vitro and in vivo.

VP40 is the matrix protein that directs budding and releases new virions from the infected cells. A recent study discovered that inhibiting the C- or N-terminal of VP40 blocks the EBOV assembly. Amino acid residues, including Thr123, His124, Phe125, Gly126, Arg134, Asn136, Tyr171, and Phe172 [18,92], are vital for docking analysis because of their interactions with RNA, of which Thr123, Phe125, and Arg134 are the most significant [93]. Similarly, the NTD residues of VP40, such as Lys127, Thr129, and Asn130, have been reported to reduce membrane localization, and the mutation of these residues critically stops the release of VLPs [24]. However, previous studies have shown that these residues overlap with the RNA-binding site, and the overlapping late (L) budding domain 7-PTAPPEY-13 contains two overlapping domains, 7-PTAP-10 and 10-PPEY-13, which have previously been shown to interact with host proteins to regulate the budding and release of VLPs [94,95]. The 7-PTAP-10 motif interacts with Tsg101, a constituent of the endosomal sorting complex crucial for transport (ESCRT-1), whereas the 10-PPEY-13 motif interacts with ubiquitin ligase (Nedd4) [95,96]. Other residues from 52–65 and 108–117 help in NTD dimerization, while _127_KATN_130_ and R134 play a role in the interaction of the NTD domain with PM, and binding of the VP40 octamer to RNA, respectively [96,97]. Other residues, including K221, k224, k25, k270, k274, and k275, present in the basic patch of CTD, are responsible for the electrostatic interaction with PM [96], while _295_LDPV_298_ is crucial for insertion of the CTD hydrophobic loop in the PM [24,96]. Our docking results show the interaction with amino acid residues in the basic patch of CTD, while several others interact within a close vicinity to _127_KATN_130_. The possible role of our compounds targeting VP40 would involve inhibition of the release of VLP and its egress as well as blocking of the interaction with PM. Similarly, small molecules have been shown to interfere with PPxY, thereby inhibiting the release of VLP in a broad range of viruses [98,99]. Another study reported that Trp191 provides stability to VP40 hydrophobic interactions and thus provides flexibility to the loop region to interact with lipids, while interfering with these residue interactions leads to dimer instability [100]. The analysis also indicated a higher probability of small molecule interactions within this vicinity [43,44,45]. Our research regarding binding site prediction using CASTp (Table S1) agrees with previous studies. Initial studies using in silico analysis were confirmed by experimental validation with a docking score of −4 to −5. Our results showed a minimum docking score; this could be a better inhibitor then the reported one, though experimental validation is still required.

**Figure 7:**
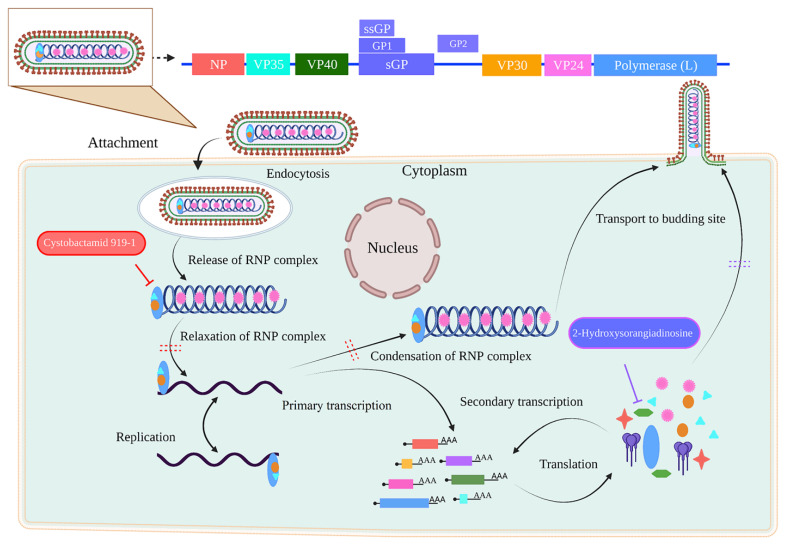
The proposed mechanism of myxobacterial natural products inhibiting the EBOV.

Molecular dynamic simulations show a protein’s absolute mobility and structural changes in its natural context. RMSD is a well-known method for the equilibration and estimation of protein stability. The RMSD results showed that the bindings of VP35_Cystobactamid 919-1 and VP40_2-Hydroxysorangiadenosine were the most stable compared to the other complexes. The flexibility of the residues was shown by RMSF, which indicated that upon binding to the identified lead compound, the protein was relatively more compact. The secondary structure results also supported our results. Protein–ligand contacts are one of the most essential components of molecular interactions in biological systems. By adding directionality and explicitness to molecular interactions, these contacts serve as the foundation for molecular recognition and selectivity. Changes in secondary structures drive protein–ligand exchanges regulated by protein–ligand connections. The lead compounds Cystobactamid 919-1 and 2-Hydroxysorangiadenosine exhibited higher contact with the ligand, indicating that these ligands were more stable within the active pockets of VP35 and VP40 proteins, respectively. The energy released during the interaction between a ligand and a protein is known as the binding energy. The total binding energy is the product of the van der Waals, electrostatic, polar solvation, and SASA energies. Except for the polar solvation energy, all other types of energies aided the interaction of various molecules with VP35 and VP40. Among all the compounds tested, the bioactive chemical Cystobactamid 934-2 and 2-Hydroxysorangiadenosine had the lowest binding free energies of −268.16 kJ/mol and −231.83 kJ/mol, respectively. Further ADME and toxicity analyses revealed that these compounds could be a possible medication against EBOV. As a result, this work reveals Cystobactamid 919-1 to be a more potent inhibitor of VP35, while 2-Hydroxysorangiadenosine is a more potent inhibitor of the VP40 protein. We provide a diagrammatic model (Figure 7) of the two myxobacterial compounds inhibiting the EBOV, which shows that targeting the VP35 and VP40 proteins can inhibit the virus at multiple stages. Furthermore, the backbone structure of Cystobactamid 919-1 and 2-Hydroxysorangiadenosine might be used to create more effective inhibitors of matrix and interferon-binding proteins.

## 5. Conclusions

This study comprehensively details a computational study of myxobacterial natural products as potential inhibitors of the EBOV VP35 and VP40 proteins, which are essential for viral replication and assembly. The analysis showed that Cystobactamid 919-1 and 2-Hydroxysorangiadenosine have high binding affinity and stability to VP35 and VP40, respectively, as confirmed by molecular docking and molecular dynamic simulations, despite minor fluctuations. These compounds also exhibit favorable pharmacological properties. Our analysis of these compounds indicated solid binding and stable interaction, suggesting that they could be further developed as novel therapeutics.

## Figures and Tables

**Table 1:** Free energy calculations for the main complexes (kJ/mol) against VP35 or VP40.

Protein	Complexes	ΔE Binding	SASA	ΔE Electrostatic	ΔE Van der Waal
VP35	Cystobactamid 934-2	−214.49	−22.22	−38.14	−289.45
	Cystobactamid 919-1	−268.16	−29.52	−317.55	−388.49
	Cittilin A	−191.14	−22.72	−98.21	−321.22
VP40	2-Hydroxysorangiadenosine	−231.83	−28.99	−189.37	−346.24
	Enhypyrazinone B	−219.20	−21.55	−32.59	−266.80
	Sorangiadenosine	−211.25	23.20	−36.68	−281.38

**Table 2:** Physicochemical properties, solubility, and pharmacokinetics of all compounds.

NPs	Physicochemical Properties (Lipinski Rule of Five)					Solubility			Pharmacokinetics	
	MW	HBD	HBA	RB	Log P	Log S (ESOL)	Log S (Ali)	Log S (SILICOS-IT)	GI Absorption	CYP Enzyme Inhibitor
Cystobactamid 934-2	934.90	8	15	25	3.89	PS	NS	NS	low	CYP2C9
Cystobactamid 919-1	917.87	8	14	23	3.44	PS	NS	NS	low	CYP2C9
Cittilin A	630.69	6	9	4	1.62	MS	MS	PS	Low	CYP3A4
2-Hydroxysorangiadenosine	487.59	5	8	5	1.7	MS	PS	S	Low	No
Enhypyrazinone B	369.46	2	2	5	4.54	MS	MS	PS	High	Yes
Sorangiadenosine	471.59	4	7	5	2.5	MS	PS	S	High	CYP2D6

Note: NS: not soluble, PS: poorly soluble, MS: moderately soluble, S: soluble.

## Data Availability

Data is contained within the article and Supplementary Materials.

## Supplementary Materials

The following supporting information can be downloaded at: https://www.mdpi.com/article/10.3390/biom14060660/s1, Figure S1. Prediction of binding sites of VP35 and VP40 proteins using CASTp. The highlighted regions represent the major binding pockets which were selected for further docking. Figure S2. Protein–ligand interaction of VP35 with Cittilin A (A), Cystobactamid 919-1 (B), and Cystobactamid 934-2 (C). Figure S3. Protein–ligand interaction of VP40 with 2-Hydroxysorangiodenosine (A), Sorangiodenosine (B), and Enhypyrazinone B (C). Table S1. Binding sites prediction of VP35 and VP40 using CASTp. Table S2. Molecular docking analysis of the main compounds against VP35 or VP40.

## References

[1] Geisbert T.W., Hensley L.E. (2004). Ebola Virus: New Insights into Disease Aetiopathology and Possible Therapeutic Interventions. Expert Rev. Mol. Med..

[2] Feldmann H., Sprecher A., Geisbert T.W. (2020). Ebola. N. Engl. J. Med..

[3] Jacob S.T., Crozier I., Fischer W.A., Hewlett A., Kraft C.S., de L. Vega M.-A., Soka M.J., Wahl V., Griffiths A., Bollinger L. (2020). Ebola Virus Disease. Nat. Rev. Dis. Primer.

[4] Aylward B., Barboza P., Bawo L., Bertherat E., Bilivogui P., Blake I., Brennan R., Briand S., Chakauya J.M., WHO Ebola Response Team (2014). Ebola Virus Disease in West Africa--the First 9 Months of the Epidemic and Forward Projections. N. Engl. J. Med..

[5] Billioux B.J., Smith B., Nath A. (2016). Neurological Complications of Ebola Virus Infection. Neurotherapeutics.

[6] MacDermott N., Herberg J.A. (2017). Ebola: Lessons Learned. Paediatr. Child Health.

[7] Redding D.W., Atkinson P.M., Cunningham A.A., Lo Iacono G., Moses L.M., Wood J.L.N., Jones K.E. (2019). Impacts of Environmental and Socio-Economic Factors on Emergence and Epidemic Potential of Ebola in Africa. Nat. Commun..

[8] Takamatsu Y., Kolesnikova L., Becker S. (2018). Ebola Virus Proteins NP, VP35, and VP24 Are Essential and Sufficient to Mediate Nucleocapsid Transport. Proc. Natl. Acad. Sci. USA.

[9] Balmith M., Soliman M.E.S. (2017). Potential Ebola Drug Targets—Filling the Gap: A Critical Step Forward towards the Design and Discovery of Potential Drugs. Biologia.

[10] Mehedi M., Falzarano D., Seebach J., Hu X., Carpenter M.S., Schnittler H.-J., Feldmann H. (2011). A New Ebola Virus Nonstructural Glycoprotein Expressed through RNA Editing. J. Virol..

[11] Takada A., Kawaoka Y. (2001). The Pathogenesis of Ebola Hemorrhagic Fever. Trends Microbiol..

[12] Olukitibi T.A., Ao Z., Mahmoudi M., Kobinger G.A., Yao X. (2019). Dendritic Cells/Macrophages-Targeting Feature of Ebola Glycoprotein and Its Potential as Immunological Facilitator for Antiviral Vaccine Approach. Microorganisms.

[13] Mühlberger E., Lötfering B., Klenk H.D., Becker S. (1998). Three of the Four Nucleocapsid Proteins of Marburg Virus, NP, VP35, and L, Are Sufficient to Mediate Replication and Transcription of Marburg Virus-Specific Monocistronic Minigenomes. J. Virol..

[14] Basler C.F., Wang X., Mühlberger E., Volchkov V., Paragas J., Klenk H.D., García-Sastre A., Palese P. (2000). The Ebola Virus VP35 Protein Functions as a Type I IFN Antagonist. Proc. Natl. Acad. Sci. USA.

[15] Basler C.F., García-Sastre A. (2002). Viruses and the Type I Interferon Antiviral System: Induction and Evasion. Int. Rev. Immunol..

[16] Haasnoot J., de Vries W., Geutjes E.-J., Prins M., de Haan P., Berkhout B. (2007). The Ebola Virus VP35 Protein Is a Suppressor of RNA Silencing. PLoS Pathog..

[17] Jasenosky L.D., Cadena C., Mire C.E., Borisevich V., Haridas V., Ranjbar S., Nambu A., Bavari S., Soloveva V., Sadukhan S. (2019). The FDA-Approved Oral Drug Nitazoxanide Amplifies Host Antiviral Responses and Inhibits Ebola Virus. iScience.

[18] Gomis-Rüth F.X., Dessen A., Timmins J., Bracher A., Kolesnikowa L., Becker S., Klenk H.D., Weissenhorn W. (2003). The Matrix Protein VP40 from Ebola Virus Octamerizes into Pore-like Structures with Specific RNA Binding Properties. Struct. Lond. Engl. 1993.

[19] Harty R.N., Brown M.E., Wang G., Huibregtse J., Hayes F.P. (2000). A PPxY Motif within the VP40 Protein of Ebola Virus Interacts Physically and Functionally with a Ubiquitin Ligase: Implications for Filovirus Budding. Proc. Natl. Acad. Sci. USA.

[20] Noda T., Sagara H., Suzuki E., Takada A., Kida H., Kawaoka Y. (2002). Ebola Virus VP40 Drives the Formation of Virus-like Filamentous Particles along with GP. J. Virol..

[21] Stahelin R.V. (2014). Membrane Binding and Bending in Ebola VP40 Assembly and Egress. Front. Microbiol..

[22] Timmins J., Scianimanico S., Schoehn G., Weissenhorn W. (2001). Vesicular Release of Ebola Virus Matrix Protein VP40. Virology.

[23] Dessen A., Volchkov V., Dolnik O., Klenk H.D., Weissenhorn W. (2000). Crystal Structure of the Matrix Protein VP40 from Ebola Virus. EMBO J..

[24] Adu-Gyamfi E., Soni S.P., Xue Y., Digman M.A., Gratton E., Stahelin R.V. (2013). The Ebola Virus Matrix Protein Penetrates into the Plasma Membrane: A Key Step in Viral Protein 40 (VP40) Oligomerization and Viral Egress. J. Biol. Chem..

[25] McCarthy S.E., Johnson R.F., Zhang Y.-A., Sunyer J.O., Harty R.N. (2007). Role for Amino Acids 212KLR214 of Ebola Virus VP40 in Assembly and Budding. J. Virol..

[26] Fabozzi G., Nabel C.S., Dolan M.A., Sullivan N.J. (2011). Ebolavirus Proteins Suppress the Effects of Small Interfering RNA by Direct Interaction with the Mammalian RNA Interference Pathway. J. Virol..

[27] Lee J.-Y., Kuo C.-J., Shin J.S., Jung E., Liang P.-H., Jung Y.-S. (2021). Identification of Non-Covalent 3C-like Protease Inhibitors against Severe Acute Respiratory Syndrome Coronavirus-2 via Virtual Screening of a Korean Compound Library. Bioorg. Med. Chem. Lett..

[28] Hansen F., Feldmann H., Jarvis M.A. (2021). Targeting Ebola Virus Replication through Pharmaceutical Intervention. Expert Opin. Investig. Drugs.

[29] Markham A. (2021). REGN-EB3: First Approval. Drugs.

[30] Saxena D., Kaul G., Dasgupta A., Chopra S. (2021). Atoltivimab/Maftivimab/Odesivimab (Inmazeb) Combination to Treat Infection Caused by Zaire Ebolavirus. Drugs Today Barc. Spain 1998.

[31] Lane T., Anantpadma M., Freundlich J.S., Davey R.A., Madrid P.B., Ekins S. (2019). The Natural Product Eugenol Is an Inhibitor of the Ebola Virus In Vitro. Pharm. Res..

[32] Reichenbach H. (2001). Myxobacteria, Producers of Novel Bioactive Substances. J. Ind. Microbiol. Biotechnol..

[33] Gerth K., Pradella S., Perlova O., Beyer S., Müller R. (2003). Myxobacteria: Proficient Producers of Novel Natural Products with Various Biological Activities--Past and Future Biotechnological Aspects with the Focus on the Genus Sorangium. J. Biotechnol..

[34] Mohr K.I., Moradi A., Glaeser S.P., Kämpfer P., Gemperlein K., Nübel U., Schumann P., Müller R., Wink J. (2018). Nannocystis Konarekensis Sp. Nov., a Novel Myxobacterium from an Iranian Desert. Int. J. Syst. Evol. Microbiol..

[35] Herrmann J., Fayad A.A., Müller R. (2017). Natural Products from Myxobacteria: Novel Metabolites and Bioactivities. Nat. Prod. Rep..

[36] Weissman K.J., Müller R. (2010). Myxobacterial Secondary Metabolites: Bioactivities and Modes-of-Action. Nat. Prod. Rep..

[37] Wenzel S.C., Müller R. (2009). The Impact of Genomics on the Exploitation of the Myxobacterial Secondary Metabolome. Nat. Prod. Rep..

[38] Hüttel S., Testolin G., Herrmann J., Planke T., Gille F., Moreno M., Stadler M., Brönstrup M., Kirschning A., Müller R. (2017). Discovery and Total Synthesis of Natural Cystobactamid Derivatives with Superior Activity against Gram-Negative Pathogens. Angew. Chem. Int. Ed Engl..

[39] Koutsoudakis G., Romero-Brey I., Berger C., Pérez-Vilaró G., Monteiro Perin P., Vondran F.W.R., Kalesse M., Harmrolfs K., Müller R., Martinez J.P. (2015). Soraphen A: A Broad-Spectrum Antiviral Natural Product with Potent Anti-Hepatitis C Virus Activity. J. Hepatol..

[40] Beck S., Henß L., Weidner T., Herrmann J., Müller R., Chao Y.-K., Grimm C., Weber C., Sliva K., Schnierle B.S. (2016). Identification of Entry Inhibitors of Ebola Virus Pseudotyped Vectors from a Myxobacterial Compound Library. Antiviral Res..

[41] Burley S.K., Bhikadiya C., Bi C., Bittrich S., Chen L., Crichlow G.V., Christie C.H., Dalenberg K., Di Costanzo L., Duarte J.M. (2021). RCSB Protein Data Bank: Powerful New Tools for Exploring 3D Structures of Biological Macromolecules for Basic and Applied Research and Education in Fundamental Biology, Biomedicine, Biotechnology, Bioengineering and Energy Sciences. Nucleic Acids Res..

[42] Rose P.W., Prlić A., Altunkaya A., Bi C., Bradley A.R., Christie C.H., Costanzo L.D., Duarte J.M., Dutta S., Feng Z. (2017). The RCSB Protein Data Bank: Integrative View of Protein, Gene and 3D Structural Information. Nucleic Acids Res..

[43] Roy A., Kucukural A., Zhang Y. (2010). I-TASSER: A Unified Platform for Automated Protein Structure and Function Prediction. Nat. Protoc..

[44] Yang J., Zhang Y. (2015). I-TASSER Server: New Development for Protein Structure and Function Predictions. Nucleic Acids Res..

[45] Zhang Y. (2008). I-TASSER Server for Protein 3D Structure Prediction. BMC Bioinform..

[46] Waterhouse A., Bertoni M., Bienert S., Studer G., Tauriello G., Gumienny R., Heer F.T., de Beer T.A.P., Rempfer C., Bordoli L. (2018). SWISS-MODEL: Homology Modelling of Protein Structures and Complexes. Nucleic Acids Res..

[47] Willard L. (2003). VADAR: A Web Server for Quantitative Evaluation of Protein Structure Quality. Nucleic Acids Res..

[48] Kim S., Chen J., Cheng T., Gindulyte A., He J., He S., Li Q., Shoemaker B.A., Thiessen P.A., Yu B. (2021). PubChem in 2021: New Data Content and Improved Web Interfaces. Nucleic Acids Res..

[49] Cousins K.R. (2005). ChemDraw Ultra 9.0. CambridgeSoft, 100 CambridgePark Drive, Cambridge, MA 02140. Www. Cambridgesoft.Com. See Web Site for Pricing Options. J. Am. Chem. Soc..

[50] Hanwell M.D., Curtis D.E., Lonie D.C., Vandermeersch T., Zurek E., Hutchison G.R. (2012). Avogadro: An Advanced Semantic Chemical Editor, Visualization, and Analysis Platform. J. Cheminformatics.

[51] Tian W., Chen C., Lei X., Zhao J., Liang J. (2018). CASTp 3.0: Computed Atlas of Surface Topography of Proteins. Nucleic Acids Res..

[52] Trott O., Olson A.J. (2010). AutoDock Vina: Improving the Speed and Accuracy of Docking with a New Scoring Function, Efficient Optimization, and Multithreading. J. Comput. Chem..

[53] Bowers K.J., Chow D.E., Xu H., Dror R.O., Eastwood M.P., Gregersen B.A., Klepeis J.L., Kolossvary I., Moraes M.A., Sacerdoti F.D. (2006). Scalable Algorithms for Molecular Dynamics Simulations on Commodity Clusters. Proceedings of the ACM/IEEE SC 2006 Conference (SC’06).

[54] Jorgensen W.L., Maxwell D.S., Tirado-Rives J. (1996). Development and Testing of the OPLS All-Atom Force Field on Conformational Energetics and Properties of Organic Liquids. J. Am. Chem. Soc..

[55] Jorgensen W.L., Chandrasekhar J., Madura J.D., Impey R.W., Klein M.L. (1983). Comparison of Simple Potential Functions for Simulating Liquid Water. J. Chem. Phys..

[56] Shaw D.E. (2005). A Fast, Scalable Method for the Parallel Evaluation of Distance-Limited Pairwise Particle Interactions. J. Comput. Chem..

[57] Hoover W.G. (1985). Canonical Dynamics: Equilibrium Phase-Space Distributions. Phys. Rev. Gen. Phys..

[58] Martyna G.J., Tobias D.J., Klein M.L. (1994). Constant Pressure Molecular Dynamics Algorithms. J. Chem. Phys..

[59] Daina A., Michielin O., Zoete V. (2017). SwissADME: A Free Web Tool to Evaluate Pharmacokinetics, Drug-Likeness and Medicinal Chemistry Friendliness of Small Molecules. Sci. Rep..

[60] Tsai H.-H.G., Tsai C.-J., Ma B., Nussinov R. (2009). In Silico Protein Design by Combinatorial Assembly of Protein Building Blocks. Protein Sci..

[61] Pettersen E.F., Goddard T.D., Huang C.C., Couch G.S., Greenblatt D.M., Meng E.C., Ferrin T.E. (2004). UCSF Chimera? A Visualization System for Exploratory Research and Analysis. J. Comput. Chem..

[62] Nasution M.A.F., Alkaff A.H., Tambunan U.S.F. (2018). Discovery of Indonesian Natural Products as Potential Inhibitor of Ebola Virus VP40 through Molecular Docking Simulation.

[63] Yu W., MacKerell A.D. (2017). Computer-Aided Drug Design Methods. Methods Mol. Biol. Clifton NJ.

[64] Ferreira L.G., Dos Santos R.N., Oliva G., Andricopulo A.D. (2015). Molecular Docking and Structure-Based Drug Design Strategies. Molecules.

[65] Hildebrand P.W., Rose A.S., Tiemann J.K.S. (2019). Bringing Molecular Dynamics Simulation Data into View. Trends Biochem. Sci..

[66] Rasheed M.A., Iqbal M.N., Saddick S., Ali I., Khan F.S., Kanwal S., Ahmed D., Ibrahim M., Afzal U., Awais M. (2021). Identification of Lead Compounds against Scm (Fms10) in Enterococcus Faecium Using Computer Aided Drug Designing. Life.

[67] Karlgren M., Bergström C.A.S., Wilson A.G.E. (2015). How Physicochemical Properties of Drugs Affect Their Metabolism and Clearance. New Horizons in Predictive Drug Metabolism and Pharmacokinetics.

[68] Kumari R., Kumar R., Lynn A., Open Source Drug Discovery Consortium (2014). G_mmpbsa —A GROMACS Tool for High-Throughput MM-PBSA Calculations. J. Chem. Inf. Model..

[69] Lipinski C.A., Lombardo F., Dominy B.W., Feeney P.J. (1997). Experimental and Computational Approaches to Estimate Solubility and Permeability in Drug Discovery and Development Settings. Adv. Drug Deliv. Rev..

[70] Lohit N., Singh A.K., Kumar A., Singh H., Yadav J.P., Singh K., Kumar P. (2024). Description and *In Silico* ADME Studies of US-FDA Approved Drugs orDrugs under Clinical Trial Which Violate the Lipinski’s Rule of 5. Lett. Drug Des. Discov..

[71] Testolin G., Cirnski K., Rox K., Prochnow H., Fetz V., Grandclaudon C., Mollner T., Baiyoumy A., Ritter A., Leitner C. (2020). Synthetic Studies of Cystobactamids as Antibiotics and Bacterial Imaging Carriers Lead to Compounds with High *in Vivo* Efficacy. Chem. Sci..

[72] Elston J.W.T., Cartwright C., Ndumbi P., Wright J. (2017). The Health Impact of the 2014-15 Ebola Outbreak. Public Health.

[73] Broni E., Ashley C., Adams J., Manu H., Aikins E., Okom M., Miller W.A., Wilson M.D., Kwofie S.K. (2023). Cheminformatics-Based Study Identifies Potential Ebola VP40 Inhibitors. Int. J. Mol. Sci..

[74] Mirza M., Ikram N. (2016). Integrated Computational Approach for Virtual Hit Identification against Ebola Viral Proteins VP35 and VP40. Int. J. Mol. Sci..

[75] Pleško S. (2015). In Silico Study of Plant Polyphenols’ Interactions with VP24–Ebola Virus Matrix Protein. Acta Chim. Slov..

[76] Raj U., Varadwaj P.K. (2016). Flavonoids as Multi-Target Inhibitors for Proteins Associated with Ebola Virus: In Silico Discovery Using Virtual Screening and Molecular Docking Studies. Interdiscip. Sci. Comput. Life Sci..

[77] Kashyap S. (2019). Comparative insillico studies on phytochemicals of ocimum as natural inhibitors of ebola vp-35 protein. Indo Am. J. Pharm. Sci..

[78] Ekins S., Freundlich J.S., Coffee M. (2014). A Common Feature Pharmacophore for FDA-Approved Drugs Inhibiting the Ebola Virus. F1000Research.

[79] Leung D.W., Borek D., Luthra P., Binning J.M., Anantpadma M., Liu G., Harvey I.B., Su Z., Endlich-Frazier A., Pan J. (2015). An Intrinsically Disordered Peptide from Ebola Virus VP35 Controls Viral RNA Synthesis by Modulating Nucleoprotein-RNA Interactions. Cell Rep..

[80] Shu T., Gan T., Bai P., Wang X., Qian Q., Zhou H., Cheng Q., Qiu Y., Yin L., Zhong J. (2019). Ebola Virus VP35 Has Novel NTPase and Helicase-like Activities. Nucleic Acids Res..

[81] Banerjee A., Mitra P. (2020). Ebola Virus VP35 Protein: Modeling of the Tetrameric Structure and an Analysis of Its Interaction with Human PKR. J. Proteome Res..

[82] Brown C.S., Lee M.S., Leung D.W., Wang T., Xu W., Luthra P., Anantpadma M., Shabman R.S., Melito L.M., MacMillan K.S. (2014). In Silico Derived Small Molecules Bind the Filovirus VP35 Protein and Inhibit Its Polymerase Cofactor Activity. J. Mol. Biol..

[83] Dilley K.A., Voorhies A.A., Luthra P., Puri V., Stockwell T.B., Lorenzi H., Basler C.F., Shabman R.S. (2017). The Ebola Virus VP35 Protein Binds Viral Immunostimulatory and Host RNAs Identified through Deep Sequencing. PLoS ONE.

[84] Prins K.C., Binning J.M., Shabman R.S., Leung D.W., Amarasinghe G.K., Basler C.F. (2010). Basic Residues within the Ebolavirus VP35 Protein Are Required for Its Viral Polymerase Cofactor Function. J. Virol..

[85] Kimberlin C.R., Bornholdt Z.A., Li S., Woods V.L., MacRae I.J., Saphire E.O. (2010). *Ebolavirus* VP35 Uses a Bimodal Strategy to Bind dsRNA for Innate Immune Suppression. Proc. Natl. Acad. Sci. USA.

[86] Leung D.W., Ginder N.D., Fulton D.B., Nix J., Basler C.F., Honzatko R.B., Amarasinghe G.K. (2009). Structure of the Ebola VP35 Interferon Inhibitory Domain. Proc. Natl. Acad. Sci. USA.

[87] Leung D.W., Prins K.C., Borek D.M., Farahbakhsh M., Tufariello J.M., Ramanan P., Nix J.C., Helgeson L.A., Otwinowski Z., Honzatko R.B. (2010). Structural Basis for dsRNA Recognition and Interferon Antagonism by Ebola VP35. Nat. Struct. Mol. Biol..

[88] Darko L.K.S., Broni E., Amuzu D.S.Y., Wilson M.D., Parry C.S., Kwofie S.K. (2021). Computational Study on Potential Novel Anti-Ebola Virus Protein VP35 Natural Compounds. Biomedicines.

[89] Glanzer J.G., Byrne B.M., McCoy A.M., James B.J., Frank J.D., Oakley G.G. (2016). In Silico and in Vitro Methods to Identify Ebola Virus VP35-dsRNA Inhibitors. Bioorg. Med. Chem..

[90] Corona A., Fanunza E., Salata C., Morwitzer M.J., Distinto S., Zinzula L., Sanna C., Frau A., Daino G.L., Quartu M. (2022). Cynarin Blocks Ebola Virus Replication by Counteracting VP35 Inhibition of Interferon-Beta Production. Antiviral Res..

[91] Daino G.L., Frau A., Sanna C., Rigano D., Distinto S., Madau V., Esposito F., Fanunza E., Bianco G., Taglialatela-Scafati O. (2018). Identification of Myricetin as an Ebola Virus VP35–Double-Stranded RNA Interaction Inhibitor through a Novel Fluorescence-Based Assay. Biochemistry.

[92] Geisbert T.W., Jahrling P.B. (1995). Differentiation of Filoviruses by Electron Microscopy. Virus Res..

[93] Hoenen T., Volchkov V., Kolesnikova L., Mittler E., Timmins J., Ottmann M., Reynard O., Becker S., Weissenhorn W. (2005). VP40 Octamers Are Essential for Ebola Virus Replication. J. Virol..

[94] Han Z., Ruthel G., Dash S., Berry C.T., Freedman B.D., Harty R.N., Shtanko O. (2020). Angiomotin Regulates Budding and Spread of Ebola Virus. J. Biol. Chem..

[95] Licata J.M., Simpson-Holley M., Wright N.T., Han Z., Paragas J., Harty R.N. (2003). Overlapping Motifs (PTAP and PPEY) within the Ebola Virus VP40 Protein Function Independently as Late Budding Domains: Involvement of Host Proteins TSG101 and VPS-4. J. Virol..

[96] Bornholdt Z.A., Noda T., Abelson D.M., Halfmann P., Wood M.R., Kawaoka Y., Saphire E.O. (2013). Structural Rearrangement of Ebola Virus VP40 Begets Multiple Functions in the Virus Life Cycle. Cell.

[97] Adu-Gyamfi E., Soni S., Jee C., Digman M., Gratton E., Stahelin R. (2014). A Loop Region in the N-Terminal Domain of Ebola Virus VP40 Is Important in Viral Assembly, Budding, and Egress. Viruses.

[98] Han Z., Lu J., Liu Y., Davis B., Lee M.S., Olson M.A., Ruthel G., Freedman B.D., Schnell M.J., Wrobel J.E. (2014). Small-Molecule Probes Targeting the Viral PPxY-Host Nedd4 Interface Block Egress of a Broad Range of RNA Viruses. J. Virol..

[99] Iglesias-Bexiga M., Palencia A., Corbi-Verge C., Martin-Malpartida P., Blanco F.J., Macias M.J., Cobos E.S., Luque I. (2019). Binding Site Plasticity in Viral PPxY Late Domain Recognition by the Third WW Domain of Human NEDD4. Sci. Rep..

[100] Johnson K., Pokhrel R., Budicini M., Gerstman B., Chapagain P., Stahelin R. (2020). A Conserved Tryptophan in the Ebola Virus Matrix Protein C-Terminal Domain Is Required for Efficient Virus-Like Particle Formation. Pathogens.

