# Supplementary material for: Identification of Prospective Ebola Virus VP35 and VP40 Protein Inhibitors from Myxobacterial Natural Products
Source: Biomolecules. 2024 Jun 5;14(6):660. doi: 10.3390/biom14060660 (PMC11201620; doi:10.3390/biom14060660)
Supplement: Supplementary file 1 [file biomolecules-14-00660-s001.zip › biomolecules-3027047-supplementary.pdf]

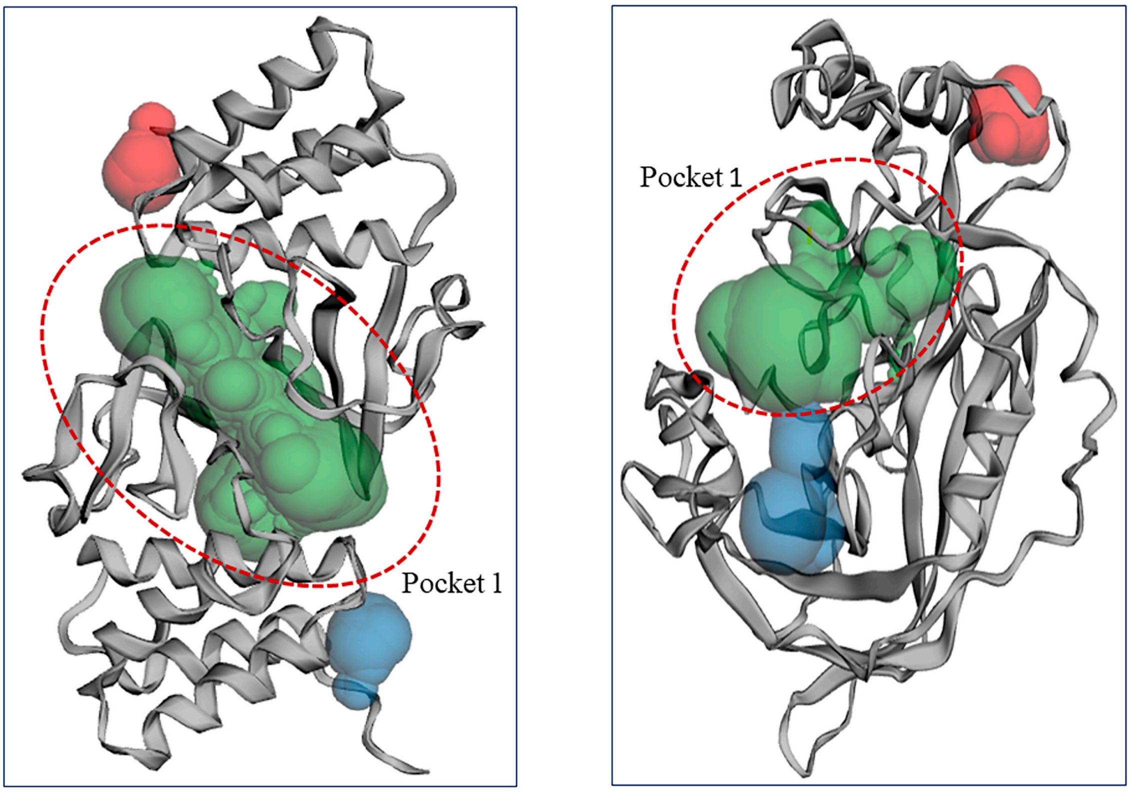

VP35, PDB:ID, 3FKE **VP40, remodeled using I-TASSAR**

Figure S1. Prediction of binding site of VP35 and VP40 proteins using CASTp. The highlighted regions represent the major binding pockets which were selected for further docking analysis.

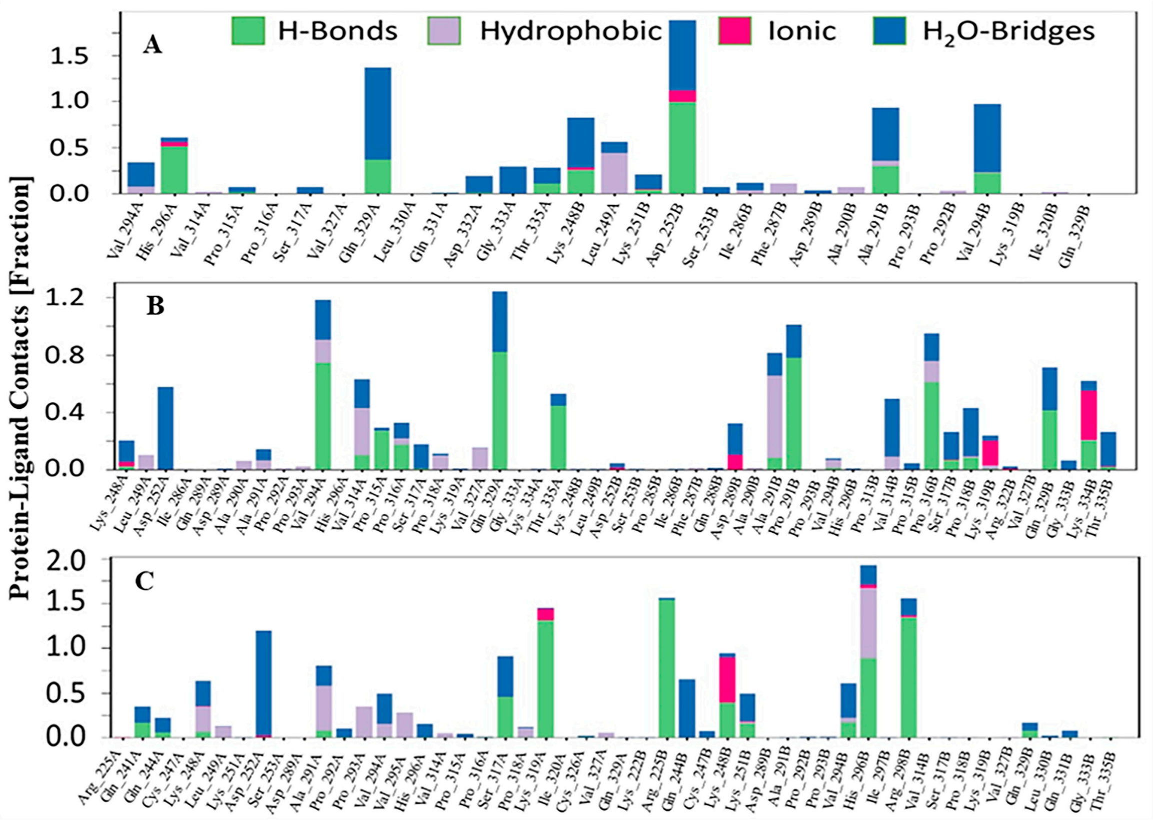

Figure S2. Protein-ligand interaction the of VP35 with Cittilin A (**A**), Cystobactamid 919-1 (**B**) and Cystobactamid 934-2 (**C**).

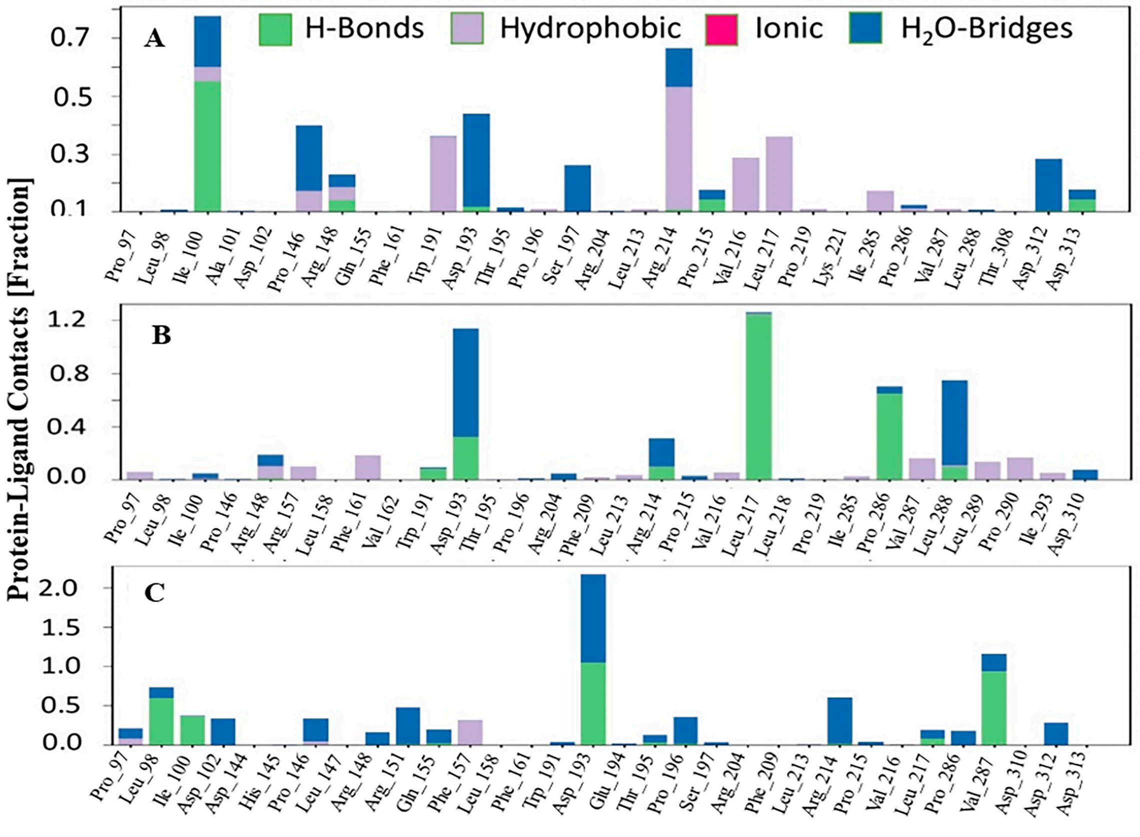

Figure S3. Protein-ligand interaction the of VP40 with 2-Hydroxysorangiodenosine (**A**), Sorangiodenosine (**B**) and Enhypyrazinone B (**C**).

Table S1. Binding site prediction of VP35 and VP40 using CASTp.

Protein	Pocket	Amino Acid residues	Area (SA) Å2	Volume (SA) Å3
VP35	Pocket 1	Val245;A, Lys248;A, Leu249;A, Asp252;A, Ser253;A, Ile286;A, Phe287;A, Gln288;A, Asp289;A, Ala290;A, Ala291;A, Pro292;A, Pro293;A, Val294;A, Ile295;A, His296;A, Ile297;A, Arg298;A, Val314;A, Pro315;A, Pro316;A, Ser317;A, Pro318;A, Lys319;A, Val327;A, Gln329;A, Leu330;A, Gln331;A, Gly333;A, Thr335;A Gln241;B, Gln244;B, Val245;B, Lys248;B, Leu249;B, Asp252;B, Ser253;B, Ile286;B, Gln288;B, Asp289;B, Ala290;B, Ala291;B, Pro292;B, Pro293;B, Val294;B, Ile295;B, His296;B, Val314;B, Pro315;B, Pro316;B, Ser317;B, Pro318;B, Lys319;B, Val327;B, Gln329;B, Leu330;B, Gln331;B, Gly333;B, Lys334;B, Thr335;B.	1155.095	1078.689
	Pocket 2	Asp218;B, Ile219;B, Asn254;B, Leu256;B, Asp257;B	48.092	35.916
	Pocket 3	Asp218;A, Ile219;A, Asn254;A, Leu256;A, Asp257;A	52.04	34.782
VP40	Pocket 1	Lys26;A, Ser27;A, Ile34;A, Gln36;A, Lys37;A, Leu98;A, Gly99;A, Ile100;A, Asp102;A, Lys104;A, Thr105;A, Ile142;A, Pro143;A, Asp144;A, His145;A, Pro146;A, Leu147;A, Arg148;A, Arg151;A, Met152;A, Trp191;A, Thr192;A, Asp193;A, Glu194;A, Thr195;A, Pro196;A, Ser197;A, Asn198;A, Leu199;A, Ser200;A,	489.047	298.67

	Ala202;A, Arg204;A, Leu217;A, Pro219;A, Ile285;A, ro286;A, Thr308;A, Asp309;A, Asp310;A, tyr311;A, Asp312;A, Asp313, Cys314;A, His315;A, Tyr322;A, Ser324;A, Lys326;A		
Pocket 2	Thr5;A, Val6;A, Ile16;A, Gln36;A, Lys37;A, Arg134, Asn136, Arg137;A, Leu138;A, Gly139;A, Gly141;A, Arg151;A, Met152;A, Gly153;A; Asn154;A	80.671	59.57
Pocket 3	Trp191;A, Glu194;A, Glu265, Val268;A, His269;A, Thr272, Lys274;A, Lys25;A, Met276;A, Ser277;A, Gln278;A, Lys279;A, Asn280;A, Gly281;A, Gln282;A, Pro283;A	118.299	52.433

Table S2. Molecular docking analysis of the main compounds against VP35 or VP40.

Target	Ligand	Docking score (kcal/mol)	Hydrogen bonds number	Hydrogen bonds and Length (Å)	Hydrophobic interactions
VP35	Cystobactamid 934-2	-10.5	6	Arg225;A (2.65), Gln241;A (2.26), Ala291;A (2.14), Val294;A (2.42, 2.31), Asp289;B (1.96)	Pro293;A, Ile295;A, Pro318;A
	Cystobactamid 919-1	-10.0	11	Asp289;A (2.62), Ala291;A (2.1, 2.03, 2.67), Ser317;A (2.08), Gln329;A (2.58), Ala291;B (2.51, 2.36), Val294;B (2.84), Lys319;B (5.47), Gln329;B (2.05)	Leu249;A, Pro292;A, Ala290;A, Val314;A, Thr335;A, Ala290;B, Pro293;B, Pro316;B
	Cittilin A	-9.8	2	Pro315;A (1.99), Ser317;A (2.64)	Pro316;A, Asp289;B, Ala290;B, Ala291;B, Val294;A, Val327, A, Val294;B
VP40	2-Hydroxysorangiadenosine	-10.9	4	Asp193;A (2.34), Thr195;A (2.4), Arg204;A (5.58), Leu217;A (1.95)	Pro97;A, Arg148;APhe157;A, Pro196;A, Arg214;A, Pro215;A, Leu288;A, Pro317;A
	Enhypyrazinone B	-10.3	2	Leu98;A (2.71), ALA156;A (2.74)	Arg148;A, Gln155;A, Phe157;A, Arg214;A, Val216;A, Val287;A, Pro290;A, Pro317;A
	Sorangiadenosine	-8.6	3	Arg148;A (2.72), Asp193;A (2.22), Leu217,A (2.54)	Phe157;A, Leu213;A, Arg214;A, Val216;A, Val 287;A, Pro317;A,
